# Maternal-Fetal Outcomes and Antibody Transfer, Depending on the Trimester of SARS-CoV-2 Infection in Non-Vaccinated Women—A Danish Nationwide Prospective Cohort Study

**DOI:** 10.3390/ijms26062533

**Published:** 2025-03-12

**Authors:** Line Fich, Ann-Marie Hellerung Christiansen, Kathrine Vauvert R. Hviid, Anna J. M. Aabakke, Eva Hoffmann, Andreas Ingham, Joaquim Ollé-López, Judith Bello-Rodríguez, Helle Gybel Juul-Larsen, Louise Kelstrup, Kathrine Perslev, Tine Dalsgaard Clausen, Line Rode, Christina Vinter, Gitte Hedermann, Marianne Jenlev Vestgaard, Richard Farlie, Anne Sørensen, Iben Sundtoft, Anne Cathrine Godtfredsen, Lars Winter Burmester, Johanna Lindman, Elin Rosenbek Severinsen, Caroline Elisabeth Kann, Christine Bo Hansen, Mette Marie Babiel Schmidt Petersen, Pia Egerup, Anne Zedeler, Amalie Dyhrberg Boje, Marie-Louise Mathilde Friis Bertelsen, Lisbeth Prætorius, Aidan Grundtvig Kristensen, Finn Stener Jørgensen, Henrik Westh, Henrik L. Jørgensen, Nina la Cour Freiesleben, Henriette Svarre Nielsen

**Affiliations:** 1 Department of Obstetrics and Gynecology, Copenhagen University Hospital Hvidovre, 2650 Hvidovre, Denmarkhenriette.svarre.nielsen@regionh.dk (H.S.N.); 2 Department of Clinical Medicine, Faculty of Health and Medical Sciences, University of Copenhagen, 2200 Copenhagen, Denmark; 3 Department of Obstetrics and Gynecology, Copenhagen University Hospital—Holbæk, 4300 Holbæk, Denmark; 4 Department of Gynecology and Obstetrics, Copenhagen University Hospital—North Zealand, 3400 Hillerød, Denmark; 5 DNRF Center for Chromosome Stability, Department of Cellular and Molecular Medicine, Faculty of Health and Medical Sciences, University of Copenhagen, 2200 Copenhagen, Denmark; 6 Department of Clinical Research, Copenhagen University Hospital Hvidovre, 2650 Hvidovre, Denmark; 7 Department of Obstetrics and Gynecology, Herlev Hospital, 2730 Herlev, Denmark; 8 Department of Clinical Biochemistry, Copenhagen University Hospital Rigshospitalet, 2100 Copenhagen, Denmark; 9 Department of Gynecology and Obstetrics, Odense University Hospital, 5000 Odense, Denmark; 10 Steno Diabetes Center Odense, Odense University Hospital, 5000 Odense, Denmark; 11 Department of Clinical Research, University of Southern Denmark, 5230 Odense, Denmark; 12 Department of Gynecology and Obstetrics, Copenhagen University Hospital Rigshospitalet, 2100 Copenhagen, Denmark; 13 Department of Obstetrics and Gynecology, Regionshospitalet Viborg, 8800 Viborg, Denmark; 14 Department of Obstetrics and Gynecology, Aalborg University Hospital, 9000 Aalborg, Denmark; 15 Department of Clinical Medicine, Faculty of Medicine, Aalborg University, 9220 Aalborg, Denmark; 16 Department of Obstetrics and Gynecology, Regionshospital Herning, 7400 Herning, Denmark; 17 Department of Obstetrics and Gynecology, Sydvestjysk Sygehus, 6700 Esbjerg, Denmark; 18 Department of Obstetrics and Gynecology, North Denmark Regional Hospital, 9800 Hjoerring, Denmark; 19 Department of Obstetrics and Gynecology, The Fertility Clinic, Copenhagen University Hospital Hvidovre, 2650 Hvidovre, Denmark; 20 Department of Clinical Microbiology, Copenhagen University Hospital Hvidovre, 2650 Hvidovre, Denmark; 21 Fetal Medicine Unit, Department of Obstetrics and Gynecology, Copenhagen University Hospital Hvidovre, 2650 Hvidovre, Denmark; 22 Department of Clinical Biochemistry, Copenhagen University Hospital Hvidovre, 2650 Hvidovre, Denmark

**Keywords:** maternal-fetal immunology, SARS-CoV-2, COVID-19, antibodies, trimester of infection, maternal-fetal outcomes, human research, non-vaccinated

## Abstract

Passive maternal-fetal transfer of severe acute respiratory syndrome coronavirus 2 (SARS-CoV-2) antibodies has been demonstrated, whilst the degree of transfer depending on the trimester of infection is lacking. Due to neonates’ immature immune systems, this knowledge could be of interest when investigating the degree of early-life protection against SARS-CoV-2. For perinatal infections such as Rubella and Toxoplasmosis, the timing of infection related to gestational age is crucial for the severity of maternal-fetal outcomes; hence, the trimester of SARS-CoV-2 infection could potentially be crucial. So far, there is no stratification on all three trimesters of SARS-CoV-2 infection in relation to maternal antibody levels in SARS-CoV-2 positive women, and the degree of transfer of SARS-CoV-2 antibodies to the newborn nor on obstetric and neonatal outcomes, which we examined in this study. Eleven departments in Denmark invited women who tested SARS-CoV-2 positive during pregnancy to participate with a blood sample and a cord blood sample at delivery. 459 SARS-CoV-2 positive women and 2567 SARS-CoV-2 negative women were included. A percentage of 87.5%, 95.3%, and 60.3% of newborns of women who tested positive in their first, second, and third trimester, respectively, had a significantly higher immunoglobin G (IgG) antibody level than their mother at delivery, indicating that the fetus is able to concentrate antibody levels or maintain the level of IgG antibodies transferred. None of the examined maternal-fetal outcomes were increased in women infected with SARS-CoV-2.

## 1. Introduction

The severe acute respiratory syndrome coronavirus-2 (SARS-CoV-2) pandemic has cost more than six million lives worldwide [1]. Many studies have investigated obstetric and neonatal outcomes in women infected with SARS-CoV-2 during pregnancy, with varying results. Although some find no differences in outcomes [2,3,4,5], others report increased risks of cesarean delivery, preterm birth, maternal death, and preeclampsia [6,7,8,9,10]. Most studies have either grouped women together without distinguishing between trimesters or focused on infection occurring during a single trimester or across two trimesters, with the majority reporting on infection in the second and third trimesters. For severe perinatal infections, such as Rubella and Toxoplasmosis, the time of infection in relation to gestational age is crucial for the severity of obstetric and neonatal outcomes. Therefore, the classification of all three trimesters of SARS-CoV-2 infection in relation to obstetric and neonatal outcomes, as well as maternal-fetal antibody transfers, could be of importance. Fallach et al. investigated two outcomes for all three trimesters: preterm birth (PTB) and small for gestational age (SGA). They found that women infected in the third trimester had an increased risk of PTB and no association of PTB if infected with SARS-CoV-2 in the first or second trimester. The risk of SGA did not differ depending on the trimester of infection [11]. Another study classified groups as SARS-CoV-2 infected <16 weeks of gestation, 16 to 28, and from 28 to 42 weeks of gestation. Although it was not possible to distinguish how many people in group one were infected in the first trimester and how many were infected during the first weeks of the second trimester up until week 16, their findings are interesting. The authors reported that maternal-fetal SARS-CoV-2 immunoglobin G (IgG) transfer seemed to be higher when SARS-CoV-2 infection occurred during the first and second trimesters [12]. Maternal-fetal transfer of IgG depends on the presence of the neonatal Fc receptor (FcRn), which is required for IgG placental crossing. The amount of FcRn expression in the placenta changes throughout the pregnancy. It is least expressed in the first trimester, increasing during the second trimester and reaching the highest expression in the third trimester [13,14,15]. Lazano et al. found that maternal-fetal IgG transfer was positively correlated with the expression of FcRn in the placenta throughout pregnancy. For several perinatal pathogens such as measles, mumps, rubella influenza, Clostridium tetani toxoid, Corynebacterium diphtheriae toxoid, and Bordetella pertussis toxin, it has been shown that mean IgG antibody levels in cord blood exceeded mean maternal IgG antibody level [16]. This aligns with the findings in a 2020 meta-analysis assessing the IgG transfer for several pathogens, which found that cord blood IgG levels, in general, were higher than maternal IgG levels [17]. Maternal-fetal SARS-CoV-2 IgG transfer has been shown in mounting numbers of studies. Several studies have shown that a passive transfer of SARS-CoV-2 antibodies takes place from woman to fetus when infection occurs during the second and third trimesters [2,18,19,20,21,22]. However, knowledge of the level of maternal-fetal transmission of SARS-CoV-2 antibodies after maternal infection in the first trimester is scarce in the current literature. Knowledge of the maternal-fetal antibody transmission pattern is valuable for the basic understanding of natural SARS-CoV-2 infection in pregnancy and could be of great interest when investigating the expected degree of early life protection against SARS-CoV-2. The objective of this study was, according to the trimester of SARS-CoV-2 infection in pregnancy, (1) To examine the maternal antibody levels of SARS-CoV-2 in positive women and the degree of transfer of SARS-CoV-2 antibodies to the newborn represented in umbilical cord blood at delivery; and (2) To examine obstetric and neonatal outcome.

## 2. Results

**Figure 1:**
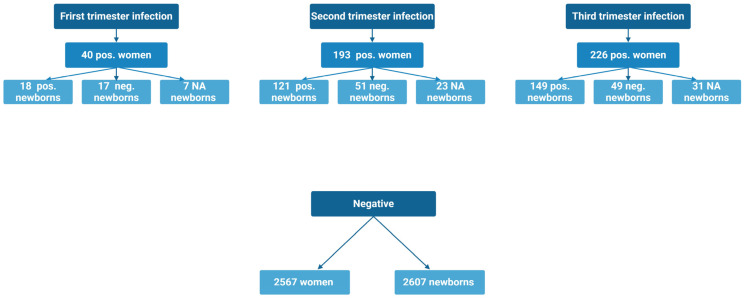
Flowchart of the included positive and negative women and their children. SARS-CoV-2 positive women were divided based on time of infection. In the first trimester, two mothers gave birth to pos/neg twins. In the second trimester, two mothers gave birth to pos/neg twins. In the third trimester, three positive newborns participated with a blood sample without their mothers and had a serology test drawn at delivery.

In total, the study included 3026 women, 459 SARS-CoV-2 positive women infected during their pregnancy, and 2567 SARS-CoV-2 IgG antibody negative women (Figure 1).

### 2.1. Characteristics Based on Trimester of Infection and SARS-CoV-2 Positive vs. Negative (Table 1 and Table 2)

Of the 459 SARS-CoV-2-positive women, 40 (8.7%) were infected in their first trimester, 193 (42.0%) in their second trimester and 226 (49.2%) in their third trimester. None of the characteristics significantly differed when comparing the time of infection (Table 1). When comparing the SARS-CoV-2 positive women with the 2567 negative women, some of the characteristics differed (Table 2). Pre-pregnancy BMI was significantly higher in the positive group compared with the negative group (24.03 kg/m^2^, IQR 21.55:27.96 vs. 23.00 kg/m^2^, IQR 20.90:26.05 vs. *p*-value = 0.02). Opposite was the prevalence of chronic disease higher in the negative groups (764 (29.7%) vs. 73 (15.9%), *p*-value = 0.02), and they were significantly older compared to the positive women (32.33 (4.59) vs. 31.04 (4.74) years, *p*-value = 0.02).

**Table 1:** Maternal characteristics for women tested positive for SARS-CoV-2 in the first, second, or third trimester.

Characteristics	SARS-CoV-2 Pos. in 1st Trimester	SARS-CoV-2 Pos. in 2nd Trimester	SARS-CoV-2 Pos. in 3rd Trimester	*p*-Value (Bonferroni Corrected)
	n = 40	n = 193	n = 226	
Age (years)	30.70 (4.73)	30.83 (4.58)	31.28 (4.89)	1.000 ^1^
Smoking	2 (5)	7 (3.6)	8 (3.5)	
Smoking before pregnancy	2 (5)	14 (7.3)	20 (8.8)	1.000 ^4^
Chronic disease	12 (30)	29 (15)	32 (14.2)	0.750 ^3^
BMI, preconceptional (kg/m^2^)	25.46 (22.20:30.52)	23.88 (21.22:28.05)	24.05 (21.82:27.68)	1.000 ^2^

BMI, body mass index. ^1^ Based on a simple linear regression model. ^2^ Based on Kruskal–Wallis test. ^3^ Based on Chi-squared test. ^4^ Based on Fisher’s exact test.

**Table 2:** Maternal characteristics for all women who tested positive for SARS-CoV-2 during pregnancy vs. SARS-CoV-2 negative.

Characteristics	SARS-CoV-2 Negative	SARS-CoV-2 Positive	*p*-Value (Bonferroni Corrected)
	n = 2567	n = 459	
Age (years)	32.33 (4.59)	31.04 (4.74)	0.020 ^1^
Smoking	91 (3.5)	17 (3.7)	
Smoking before pregnancy	315 (12.3)	36 (7.8)	0.484 ^3^
Chronic disease *	764 (29.7)	73 (15.9)	0.020 ^3^
BMI, preconceptional(kg/m^2^)	23.00 (20.90:26.05)	24.03 (21.55:27.96)	0.020 ^2^

BMI, body mass index. ^1^ Based on a simple linear regression model. ^2^ Based on Kruskal–Wallis test. ^3^ Based on Chi-squared test. * Includes diabetes mellitus type 1 and type 2, hypertension, endometriosis, PCOS, renal insufficiency, asthma, and unspecified hematological and autoimmune conditions.

### 2.2. Vertical Transmission of SARS-CoV-2 Antibodies and Maternal-Fetal Outcomes When Infected in the First Trimester (Table 3 and Table 4)

**Figure 2:**
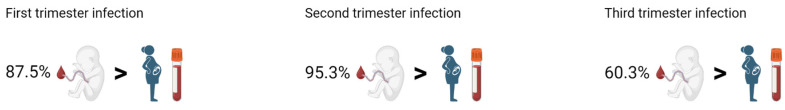
Percentage of newborns with higher IgG antibody level than their mother’s at delivery.

Of the 40 women infected in the first trimester, 21 (52.5%) had a positive PCR test, and 19 (47.5%) women were positive on their first-trimester risk assessment blood test with SARS-CoV-2 IgG antibodies ranging from 10.00 AU/mL to 100.1 AU/mL. Thirty-six women and 35 of their children participated with blood samples at the time of delivery, and 13 (36.1%) of the positive women still had IgG antibodies of 10.00 AU/mL or higher. Of their newborns, 18 (51.4%) were SARS-CoV-2 IgG antibody positive at delivery, with levels ranging from 11.00 AU/mL to 82.09 AU/mL. Seventeen (48.6%) newborns were SARS-CoV-2 IgG antibody negative, with levels ranging from 0.29 AU/mL to 9.33 AU/mL. Of the 32 umbilical cord samples available with a corresponding maternal blood sample from the time of delivery, 28 (87.5%) newborns had a significantly higher IgG antibody level than their mother (mean difference 5.85 AU/mL 95%CI (2.51–9.18 AU/mL), *p*-value < 0.001) (See Figure 2).

In Table 3, the outcomes for SARS-CoV-2-positive women are presented according to the trimester of infection. We found a significantly higher frequency of postpartum hemorrhage (PPH) in women infected in the first compared to the second and third trimester (8 (20%) vs. 13 (6.7%) vs. 11 (4.9%), *p*-value = 0.049). No other maternal-fetal or neonatal outcomes had a significant difference if infected in the first trimester compared to the other trimesters of infection (Table 3 and Table 4).

**Table 3:** Maternal outcomes for women who tested positive for SARS-CoV-2 in first, second, or third trimester.

Outcomes	SARS-CoV-2 Pos. in 1st Trimester	SARS-CoV-2 Pos. in 2nd Trimester	SARS-CoV-2 Pos. in 3rd Trimester	*p*-Value (Bonferroni Corrected)
	n = 40	n = 193	n = 226	
Gestational age at delivery (days)	276.50 (270.75:284.00)	279.00 (272.00:286.00)	281.00 (274.00:287.00)	0.758 ^2^
GDM	1 (2.5)	10 (5.2)	17 (7.5)	1.000 ^4^
Gestational hypertension	0 (0)	5 (2.6)	6 (2.7)	1.000 ^4^
Preeclampsia	0 (0)	6 (3.1)	12 (5.3)	1.000 ^4^
Preterm PROM	2 (5)	1 (0.5)	1 (0.4)	0.794 ^4^
Spontaneous labor	32 (84.2)	140 (72.5)	173 (77.6)	
Induced labor	6 (15.8)	53 (27.5)	50 (22.4)	1.000 ^3^
Vaginal delivery	29 (72.5)	162 (83.9)	183 (81)	
CS delivery	11 (27.5)	31 (16.1)	43 (19)	1.000 ^3^
Planned CS	4 (36.4)	13 (41.9)	15 (34.9)	
Emergency CS grade 3	3 (27.3)	15 (48.4)	18 (41.9)	
Emergency CS grade 2	4 (36.4)	2 (6.5)	9 (20.9)	
Emergency CS grade 1	0 (0)	1 (3.2)	1 (2.3)	1.000 ^4^
Accumulated acute CS	7 (17.5)	18 (9.3)	28 (12.4)	1.000 ^4^
Vacuum extraction	3 (7.5)	9 (4.7)	17 (7.5)	1.000 ^4^
Outcome Singleton alive	38 (95)	191 (99)	221 (97.8)	
Outcome Singleton stillbirth	0 (0)	0 (0)	2 (0.9)	
Outcome Gemelli alive	2 (5)	2 (1)	3 (1.3)	1.000 ^4^
PPH	8 (20)	13 (6.7)	11 (4.9)	**0.049 ^3^**
Any pregnancy complications *	3 (7.5)	23 (11.9)	35 (15.5)	1.000 ^3^
Liver affected	0 (0)	4 (2.1)	5 (2.2)	1.000 ^4^

GDM, gestational diabetes; PROM, prelabor rupture of membranes; CS, cesarean section; PPH, postpartum hemorrhage. Data are median (IQR) or n (%) unless otherwise specified. Emergency CS grade 3: performed within one hour from the decision, grade 2 within 30 min, grade 1 within 15 min. * Any pregnancy complications included GDM, gestational hypertension, preeclampsia, and preterm PROM. ^2^ Based on Kruskal–Wallis test. ^3^ Based on Chi-squared test. ^4^ Based on Fisher’s exact test.

**Table 4:** Neonatal outcomes for newborns of women who tested positive for SARS-CoV-2 in the first, second, or third trimester.

Characteristics/Outcomes	SARS-CoV-2 Pos. in 1st Trimester	SARS-CoV-2 Pos. in 2nd Trimester	SARS-CoV-2 Pos. in 3rd Trimester	*p*-Value (Bonferroni Corrected)
	n = 42	n = 195	n = 229	
Male	25 (59.5)	102 (52.3)	121 (52.8)	
Female	17 (40.5)	93 (47.7)	108 (47.2)	1.000 ^3^
Gestational age at delivery (days)	275.00 (270.00:284.00)	279.00 (272.00:286.00)	281.00 (274.00:287.00)	0.292 ^2^
Preterm birth ¨	4 (9.5)	7 (3.6)	12 (5.2)	1.000 ^4^
Neonatal admission	6 (14.3)	9 (4.6)	17 (7.5)	0.877 ^4^
Any neonatal complications *	10 (23.8)	19 (9.7)	30 (13.2)	0.524 ^3^
Apgar score < 7 at 5 min	1 (2.4)	1 (0.5)	3 (1.3)	1.000 ^4^
Apgar score 5 min	10.00 (10.00:10.00)	10.00 (10.00:10.00)	10.00 (10.00:10.00)	1.000 ^2^
Umbilical artery pH < 7	0 (0)	0 (0)	1 (0.4)	1.000 ^4^
Umbilical artery pH	7.25 (7.20:7.27)	7.24 (7.19:7.29)	7.23 (7.18:7.28)	1.000 ^2^
Malformation	1 (2.4)	2 (1)	5 (2.2)	1.000 ^4^
Low birth weight < 2500 g	5 (11.9)	7 (3.6)	9 (3.9)	0.979 ^4^
Birth weight (gram)	3306.19 (660.80)	3567.58 (490.37)	3555.53 (527.66)	0.133 ^1^

Data are median (IQR) or n (%) unless otherwise specified. ¨ Before gestational week 37 + 0 days. * Any neonatal complications included preterm birth, neonatal admission, 5-min Apgar score less than 7, arterial pH less than 7, malformations and low birth weight. ^1^ Based on a simple linear regression model. ^2^ Based on Kruskal–Wallis test. ^3^ Based on Chi-squared test. ^4^ Based on Fisher’s exact test.

### 2.3. Vertical Transmission of SARS-CoV-2 Antibodies and Maternal-Fetal Outcomes When Infected in the Second Trimester (Table 3 and Table 4)

A total of 193 (42.0%) women were infected with SARS-CoV-2 in their second trimester. Of the 193 women, 173 (89.6%) had a positive PCR test, and 20 (10.4%) women were positive according to their blood test taken at the second-trimester ultrasound with SARS-CoV-2 IgG antibodies ranging from 10.37 AU/mL to 97.33 AU/mL. At the time of delivery, 91 (50.3%) of the positive women had IgG antibodies of 10.00 AU/mL or higher, and 121 (70.3%) of their newborns were SARS-CoV-2 IgG antibody positive at delivery with levels ranging from 10.15 AU/mL to 131 AU/mL. Fifty-one (29.7%) newborns were SARS-CoV-2 IgG antibody negative, with levels ranging from 0.35 AU/mL to 9.97 AU/mL. Of the 169 umbilical cord samples available with a corresponding maternal blood sample at the time of delivery, 161 (95.3%) newborns had a significantly higher IgG antibody level than their mother (mean difference 14.14 AU/mL 95%CI (11.50–16.79 AU/mL), *p*-value < 0.001) (See Figure 2).

Women who tested positive in their second trimester had no significantly different maternal-fetal or neonatal outcomes compared to the other trimesters of infection (Table 3 and Table 4).

### 2.4. Vertical Transmission of SARS-CoV-2 Antibodies and Maternal-Fetal Outcomes When Infected in the Third Trimester (Table 3 and Table 4)

Of the 226 women infected in their third trimester, 199 (88.1%) women had a positive PCR test. Twenty-four (11.9%) women were considered positive according to a blood test in the third trimester with SARS-CoV-2 IgG antibodies ranging from 10.37 AU/mL to 97.33 AU/mL. Three women had negative SARS-CoV-2 IgG antibodies, but their newborns had SARS-CoV-2 IgG antibodies, and therefore the women were considered positive. At the time of delivery, 152 (73.8%) of the positive women had IgG antibodies of 10.00 AU/mL or higher, and 149 (75.3%) of their newborns were SARS-CoV-2 IgG antibody positive at delivery with levels ranging from 10.11 AU/mL to 141.3 AU/mL. Forty-nine (24.7%) newborns were SARS-CoV-2 IgG antibody negative with levels ranging from 0.11 AU/mL to 9.44 AU/mL. Of the 184 umbilical cord samples available, with a corresponding maternal blood sample at the time of delivery, 111 (60.3%) newborns had a significantly higher IgG antibody level than their mother (median 0.39 IQR −13.48:5.26, *p*-value (based on Wilcoxon signed rank test) = 0.035) (See Figure 2).

Women who tested positive in their third trimester had no significant difference in maternal-fetal or neonatal outcomes compared to the other trimesters of infection (Table 3 and Table 4).

### 2.5. Vertical Transmission of SARS-CoV-2 Antibodies and Maternal-Fetal Outcomes When Accumulating All SARS-CoV-2 Positive Women (Table 5 and Table 6)

In total, 459 women were SARS-CoV-2 positive during pregnancy, of which 66 (14%) were detected by SARS-CoV-2 serology tests, and 393 (86%) were detected by PCR tests. Of these accumulated positive women, 403 newborns (87.8%) had an umbilical cord blood sample drawn. The SARS-CoV-2 negative group consisted of 2567 women and 2607 newborns.

When accumulating all women who were SARS-CoV-2 positive during pregnancy, we found no significant differences in any maternal-fetal or neonatal outcomes if infected with SARS-CoV-2 during pregnancy compared to non-infected. We found vacuum extraction (764 (29.7%) vs. 73 (15.9%), *p*-value = 0.03) and PPH (666 (26.0%) vs. 32 (7.0%), *p*-value = 0.02) increased for the SARS-CoV-2 negative control group compared to the SARS-CoV-2 positive case group. Only 3 (0.65%) of the 459 SARS-CoV-2 positive women in our study were hospitalized due to SARS-CoV-2 infection.

Of the 403 umbilical cord blood samples, 70 newborns had both SARS-CoV-2 IgG and IgM measured, and none tested positive for SARS-CoV-2 IgM antibodies. All 403 umbilical cord blood samples were tested for the presence of IgG, and 284 (70.5%) had SARS-CoV-2 IgG antibodies ranging from 10.28 AU/mL to 113.02 AU/mL. The remaining 119 (29.5%) umbilical cord blood samples with IgG antibodies < 10.00 ranged from 0.11 AU/mL to 9.97 AU/mL, and of these newborns, six were admitted to the neonatal department, three were born before gestational week 34 + 0, four had a birthweight < 2500 g (all born before GA 34 + 0) and one newborn was stillborn.

**Table 5:** Maternal outcomes for all women tested positive for SARS-CoV-2 during pregnancy vs. SARS-CoV-2 negative.

Outcomes	SARS-CoV-2 Negative	SARS-CoV-2 Positive	*p*-Value (Bonferroni Corrected)
	n = 2567	n = 459	
Gestational age at delivery (days)	281.00 (273.00:287.00)	280.00 (273.00:286.00)	1.000 ^2^
GDM	192 (7.5)	28 (6.1)	1.000 ^3^
Gestational hypertension	91 (3.5)	11 (2.4)	1.000 ^3^
Preeclampsia	107 (4.2)	18 (3.9)	1.000 ^3^
Preterm PROM	68 (2.6)	4 (0.9)	0.658 ^3^
Spontaneous labor	1755 (75.8)	345 (76)	
Induced labor	559 (24.2)	109 (24)	1.000 ^3^
Vaginal delivery	2037 (79.4)	374 (81.5)	
CS delivery	530 (20.6)	85 (18.5)	1.000 ^3^
Planned CS	223 (42.2)	32 (37.6)	
Emergency CS grade 3	190 (35.9)	36 (42.4)	
Emergency CS grade 2	103 (19.5)	15 (17.6)	
Emergency CS grade 1	13 (2.5)	2 (2.4)	1.000 ^4^
Accumulated acute CS	306 (11.9)	53 (11.5)	1.000 ^3^
Vacuum extraction	292 (11.4)	29 (6.3)	**0.032 ^3^**
Outcome Singleton alive	2528 (98.4)	450 (98)	
Outcome Singleton stillbirth	5 (0.2)	2 (0.4)	
Outcome Gemelli alive	35 (1.4)	7 (1.5)	
Outcome Gemelli 1 alive 1 stillbirth	1 (0)	0 (0)	1.000 ^4^
PPH	666 (26)	32 (7)	**0.020 ^3^**
Any pregnancy complications *	436 (17)	61 (13.3)	1.000 ^3^
Liver affected	32 (1.2)	9 (2)	1.000 ^3^

GDM, gestational diabetes; PROM, prelabor rupture of membranes; CS, cesarean section; PPH, postpartum hemorrhage. Data are median (IQR) or n (%) unless otherwise specified. * Any pregnancy complications included GDM, gestational hypertension, preeclampsia and preterm PROM. ^2^ Based on Kruskal–Wallis test. ^3^ Based on Chi-squared test. ^4^ Based on Fisher’s exact test.

**Table 6:** Neonatal outcomes for newborns of all women who tested positive for SARS-CoV-2 during pregnancy vs. SARS-CoV-2 negative.

Characteristics/Outcomes	Newborns of SARS-CoV-2 Negative	Newborns of SARS-CoV-2 Positive	*p*-Value (Bonferroni Corrected)
	n = 2607	n = 466	
Male	1326 (50.9)	248 (53.2)	
Female	1281 (49.1)	218 (46.8)	1.000 ^3^
Gestational age at delivery	280.00 (272.00:287.00)	280.00 (273.00:286.00)	1.000 ^2^
Preterm birth ¨	141 (5.4)	23 (4.9)	1.000 ^3^
Neonatal admission	310 (11.9)	32 (6.9)	**0.024 ^3^**
Any neonatal complications *	466 (17.9)	59 (12.7)	0.088 ^3^
Apgar score < 7 at 5 min	24 (0.9)	5 (1.1)	1.000 ^4^
Apgar score 5 min	10.00 (10.00:10.00)	10.00 (10.00:10.00)	1.000 ^2^
Umbilical artery pH < 7	11 (0.4)	1 (0.2)	1.000 ^4^
Umbilical artery pH	7.24 (7.18:7.29)	7.24 (7.18:7.28)	1.000 ^2^
Malformation	61 (2.3)	8 (1.7)	1.000 ^3^
Low birth weight < 2500 g	122 (4.7)	21 (4.5)	1.000 ^3^
Birth weight (gram)	3478.19 (563.36)	3538.10 (529.82)	0.396 ^1^

Data are median (IQR) or n (%) unless otherwise specified. ¨ Before gestational week 37 + 0 days * Any neonatal complications included preterm birth, neonatal admission, 5-min Apgar score less than 7, arterial pH less than 7, malformations and low birth weight. ^1^ Based on a simple linear regression model. ^2^ Based on Kruskal–Wallis test. ^3^ Based on Chi-squared test. ^4^ Based on Fisher’s exact test.

### 2.6. Women with Multiple Blood Samples During Pregnancy

CHH was the only study site having multiple serum samples from the same positive woman. Sixteen first-trimester positive women were identified during the data collection for the negative cohort and participated as a longitudinal cohort with multiple blood samples throughout the pregnancy. Serology development during pregnancy for SARS-CoV-2 positive mothers in correlation to their newborns’ antibody level can be seen in the Supplementary Table S1 and Supplementary Figure S1.

## 3. Discussion

Our nationwide prospective cohort study included 459 women with SARS-CoV-2 infection during pregnancy and 2567 non-infected pregnant women. We found newborns of positive mothers to have a significantly higher SARS-CoV-2 IgG antibody level compared to their mothers at delivery. When accumulating all positive women, we found no higher obstetric or neonatal risks for the women infected with SARS-CoV-2 compared to the non-infected.

In total, 87.5%, 95.3%, and 60.3% of newborns of women who tested positive in their first, second, and third trimester, respectively, had higher IgG antibody levels than their mother’s at delivery.

The higher level of antibodies in the newborns’ cord blood compared to their mother’s demonstrated in this study correlates with findings of, for instance, pertussis toxin antibodies during pregnancy and aligns with general findings on maternofetal transport of immunoglobins [17,23]. After COVID-19 vaccination in different trimesters, Atyeo et al. found a higher transfer ratio of IgG titer generated by first and second-trimester vaccination compared to third-trimester vaccination. Furthermore, they found that total IgG titer after first-trimester vaccination was significantly lower than that in the cord blood of second-trimester vaccination, which aligns with our findings of IgG in the cord blood depending on the trimester of infection [24]. Neonates’ immune systems are not yet fully developed at birth, and therefore, these placental transferred SARS-CoV-2 antibodies can hopefully support the neonates’ extrauterine protection against SARS-CoV-2 infection until the maturation of their own immune systems [17]. To the best of our knowledge, no other studies have demonstrated this for SARS-CoV-2 antibody transfer depending on all trimesters of infection. This knowledge of the maternal-fetal antibody transmission pattern is valuable for the basic understanding of SARS-CoV-2 in pregnancy and for future investigation on the expected degree of early life protection against SARS-CoV-2 but could also be of interest if investigating the paradoxical concept of blunting when considering vaccination of neonates. Blunting is when maternofetal transferred antibodies influence the antibody response expected from infant vaccination and has been seen in both related and unrelated vaccinations [17,25]. None of the newborns who were tested for both IgG and IgM in our study showed IgM antibodies in their umbilical cord blood, indicating no vertical transmission, which is consistent with most previous studies [2,20,21,26]. Only a few studies have shown detectable and infrequent SARS-CoV-2 IgM antibodies in newborns of SARS-CoV-2 infected mothers [27,28]. Of women who tested positive during pregnancy, 284 (70.5%) of their newborns had IgG antibodies of ≥10.00 AU/mL, indicating passive immunization. The finding of no higher risk of obstetric or neonatal complications if infected with SARS-CoV-2 during pregnancy is confirmed in other studies [2,3,4,5,6], although some studies found an increased risk of maternal mortality and severe pregnancy complications when infected with the SARS-CoV-2 delta variant compared to the alpha or omicron variant during pregnancy [29,30,31]. The different findings may be explained by differences in SARS-CoV-2 variants or differences in population baseline, healthcare system, and government preventive strategies during the pandemic [9,10,28,29]. Compared to other studies, our study participants were young, had an average pre-pregnancy BMI < 25, were non-smokers, and only a few had chronic diseases [9]. In Denmark, the healthcare system is free of charge, and pregnant women are offered a series of ultrasound examinations as well as doctor- and midwife appointments during pregnancy. This may contribute to preventing serious outcomes, regardless of social and economic status. When the pandemic hit, Denmark quickly underwent nationwide lockdown, and pregnant women were considered a risk group out of a precautionary principle [32]. This may have prevented not only several cases but also severe cases, and notably, only 0.65% of the SARS-CoV-2-positive women in our study were hospitalized due to SARS-CoV-2 infection.

## 4. Materials and Methods

This study was designed and initiated during the first wave of the pandemic in Denmark in early spring 2020. The study consists of two prospective cohorts focusing on SARS-CoV-2 infection during pregnancy and childbirth, a positive and a negative cohort. Data for the two cohorts were collected separately (See Figure 1).

### 4.1. Positive Cohort

The positive cohort consists of women with a SARS-CoV-2 positive test during pregnancy. Women were eligible for participation if they had a positive SARS-CoV-2 PCR test, a positive antigen test, or a positive antibody test and delivered between 4 April 2020 and 31 May 2021 at one of 11 obstetric departments in Denmark, accounting for ~66% of all births in Denmark [33]. (Hvidovre Hospital, Herlev Hospital, Rigshospitalet, Odense University Hospital, Regionshospitalet Viborg, Aalborg Hospital, Regionshospital Herning, Sydvestjysk Sygehus, Esbjerg, Hjoerring Hospital, Holbæk Hospital, Hillerød Hospital). Eligible women were invited to participate with a maternal venous blood sample and an umbilical cord blood sample (mixed venous and arterial blood) from their newborn at delivery. The trimester of infection was defined according to the date of the first positive SARS-CoV-2 test. These women were included in the positive cohort.

### 4.2. Negative Cohort

The negative cohort was established during a three-month data collection period at the Department of Obstetrics and Gynecology, Copenhagen University Hospital Hvidovre (CHH). All pregnant women at CHH were invited to participate, establishing the negative cohort. If having a positive test, women were included in the positive cohort instead.

First trimester: All women with a first-trimester risk assessment blood test (taken between gestational age 8 weeks to 13 weeks and 6 days) were invited to participate with their stored blood sample, which was taken at CHH from 17 February 2020 to 23 April 2020. Women were contacted electronically with written information about the study and had to sign an electronic informed consent to participate.

Second trimester: Between 4 April and 3 July 2020, all women attending their second-trimester ultrasound at CHH were invited to participate with a blood sample. All received oral and written information.

Third trimester: Between 4 April and 3 July 2020, all women giving birth at CHH were invited to participate with a blood sample at birth and an umbilical cord blood sample from their newborn. All received oral and written information.

None of the positive or negative participants in the study were SARS-CoV-2 vaccinated.

### 4.3. Analyses

Maternal and fetal blood samples from the positive and negative cohorts were analyzed for SARS-CoV-2 antibodies using YHLO’s iFlash 1800 and SARS-CoV-2 IgM/IgG kits. IgM antibody level of 8.00 AU/mL or higher was considered positive, and levels less than 8.00 AU/mL were considered negative. IgG antibody level of 10.00 AU/mL or higher was considered positive, and less than 10.00 AU/mL was negative [34]. If a woman tested IgM antibody positive and IgG antibody negative, she was offered an extra follow-up blood sample. If this sample continued to show no development of IgG antibodies or continued to be only IgM antibody positive, the woman was considered SARS-CoV-2 negative. If she did not have an extra blood sample taken, she was also considered negative since IgM is erratic, and due to the possibility of positive IgM antibodies could be caused by a cross reaction. If she had a positive PCR test for SARS-CoV-2, the date of the first positive test was considered the date of infection (DOI). For the 63 women only positive with IgG antibodies, the DOIs were set to 14 days prior to the blood sample.

### 4.4. SARS-CoV-2 Variants

A total of 446 women were positive before the first case of Delta SARS-CoV-2 was identified in Denmark. These were most likely infected with the dominating Alpha SARS-CoV-2 variation or one of the ten most abundant pre-Alpha lineages in Denmark. The 13 remaining women tested positive in the week the first Delta case appeared in Denmark, and the Alpha variant still being the dominant one [35,36].

### 4.5. Baseline Characteristics and Outcomes

Baseline characteristics of the women enrolled in the study, as well as obstetric outcomes and neonatal outcomes, were recorded by cross-referencing with electronic medical records. For the national cohort of SARS-CoV-2 positive women, these data were stored in the Danish COVID-19 pregnancy database, which is based in EasyTrial (easytrial.net, Denmark), as described previously by Aabakke et al. [6]. Data for the negative cohort were stored in the COVID & Pregnancy Redcap database. If a pregnant woman in the negative cohort moved and gave birth at a hospital not using the EPIC electronic platform for medical records, she was considered lost to follow-up since data could no longer be accessed by the research team.

### 4.6. Statistics

Obstetric and neonatal outcomes are presented for SARS-CoV-2 positive and for SARS-CoV-2 negative women. The women who tested positive were divided into groups depending on which trimester they initially tested positive, and outcomes are presented as mean with standard deviation (SD) or median with interquartile range (IQR) for continuous variables and numbers with percentages for discrete variables. Normality was investigated using QQ-plots for visual inspection. Differences in obstetric and neonatal outcomes between positive and negative women were investigated using a simple linear regression model for continuous outcomes, a Kruskal–Wallis test for ordinal outcomes, a Chi-squared test for discrete outcomes, and a Fisher’s exact test for discrete outcomes with fewer than five observations in the cells. To account for multiple comparison testing, we used the Bonferroni correction. To test for differences in IgG antibody levels between the women and their children, we used a paired t-test and reported means and 95% confidence intervals (95%CI). If the normality of variables could not be assumed, we used the Wilcoxon Signed Rank Test and reported medians and IQR. The level of significance was set at less than 0.05. R version 3.6.1 (The R Foundation for Statistical Computing, Vienna, Austria) was used for statistical analysis and generation of figures.

## 5. Conclusions

This prospective cohort study found a strong and statistically significant tendency for newborns of SARS-CoV-2 positive mothers to have a higher IgG antibody level compared to their mother’s IgG antibody level at delivery. This indicates that the fetus is either able to concentrate antibody levels or maintain the level of IgG antibodies transferred. No higher risk of the obstetric or neonatal outcomes analyzed in this study if infected with SARS-CoV-2 during pregnancy was found. Future research should investigate the duration and neutralizing effect of the passively transferred antibodies from mother to neonate to examine the degree of antibody protection.

### Strengths and Limitations

This study has several strengths. First, the large study population with SARS-CoV-2 antibody measurements on all 3026 participating women and 3073 of their newborns. Secondly, we have a complete obstetric and neonatal medical record of all participants independent of infection status. Thirdly, we have minimalized selection bias due to approximately >95% of all births in Denmark taking place in a hospital. Lastly, as part of conducting the negative cohort at CHH, we had a participation rate of 75.1% for the first trimester, 61% for the second trimester, and 72.5% for parturient women. Limitations: The negative cohort was based on data from one hospital, but it is the largest birthplace in Denmark (annually 11–12% of all births in Denmark), covering a geographic area with a large variation in socio-economic status [37,38]. Approximately 60% of obstetric departments in Denmark participated in the study. With only 40 women being SARS-CoV-2 positive in their first trimester, the analysis in this group should be taken with caution due to the low number. Other limitations are the 56 newborns of positive women who did not have an umbilical cord blood sample taken, primarily missing due to PPH, acute cesarean section, blood sample coagulation, or rush time at the delivery wards. This could, of course, bias the results.

## Data Availability

Data are contained within the article or Supplementary Material.

## Supplementary Materials

The following supporting information can be downloaded at: https://www.mdpi.com/article/10.3390/ijms26062533/s1.
