# Supplementary material for: Maternal-Fetal Outcomes and Antibody Transfer, Depending on the Trimester of SARS-CoV-2 Infection in Non-Vaccinated Women—A Danish Nationwide Prospective Cohort Study
Source: Int J Mol Sci. 2025 Mar 12;26(6):2533. doi: 10.3390/ijms26062533 (PMC11942278; doi:10.3390/ijms26062533)
Supplement: Supplementary file 1 [file ijms-26-02533-s001.zip › ijms-3480049-supplementary.pdf]

Maternal-fetal outcomes and antibody transfer, depending on the trimester of SARS-CoV-2 infection in non-vaccinated women – A Danish nationwide prospective cohort study.

Supplementary material:

Table S1. Maternal & cord blood serology for 16 women infected in their first trimester, with multiple blood samples during pregnancy.

IgM/IgG First trimester Mother	IgM/IgG W. 20 Scan Mother	IgM/IgG Birth Mother	IgM/IgG Birth Child (Gemelli 2)
**IgG Positive**			
**1.49/49.72**	NA	0,64/39.28	0.41/42.19
**0.74/18.34**	0.64/13.72	0.56/7.52	0.21/18.19
**0.78/22.06**	1.25/12.49	0.53/13.1	0.28/17.37 (0.25/16.36)
**3.08/100.1**	2.5/38.8	1.53/4.98	0.23/13.83
**0.25/10.11**	0.18/9.48	0.39/6.12	0.26/11.16
**1.74/30.7**	2.63/10.05	1.45/4.79	0.29/9.33
**0.47/10.00**	0.55/8.9	0,78/0.27	0.25/8.77
**30.42/45.88**	3.69/48.86	4.89/4.76	0.17/8.64
**0.91/10.73**	0.97/14.03	0,6/8.96	0.29/5.75 (0.28/6.56)
**0.87/26.86**	0.54/8.83	0,53/1.94	0.25/4.06
**2.09/10.00**	NA	0,74/1.81	0.38/3.73
**1.15/21.07**	1.1/12.93	1,1/2.69	0.27/3.6
**8.87/49.51**	4.6/51.4	4.02/4.95	0.18/0.58
**0.96/10.32**	0.99/8.08	1,09/63.69	NA
**0.47/10.35**	0.43/10.3	0,54/6.79	NA
**0.29/11.26**	0.29/6.85	NA	NA

NA, not available.

The table illustrates that some cases show an unpredictable development of maternal antibodies.

Figure S1. Serology development during pregnancy for SARS-CoV-2 positive mothers in correlation to their newborns' antibody level.

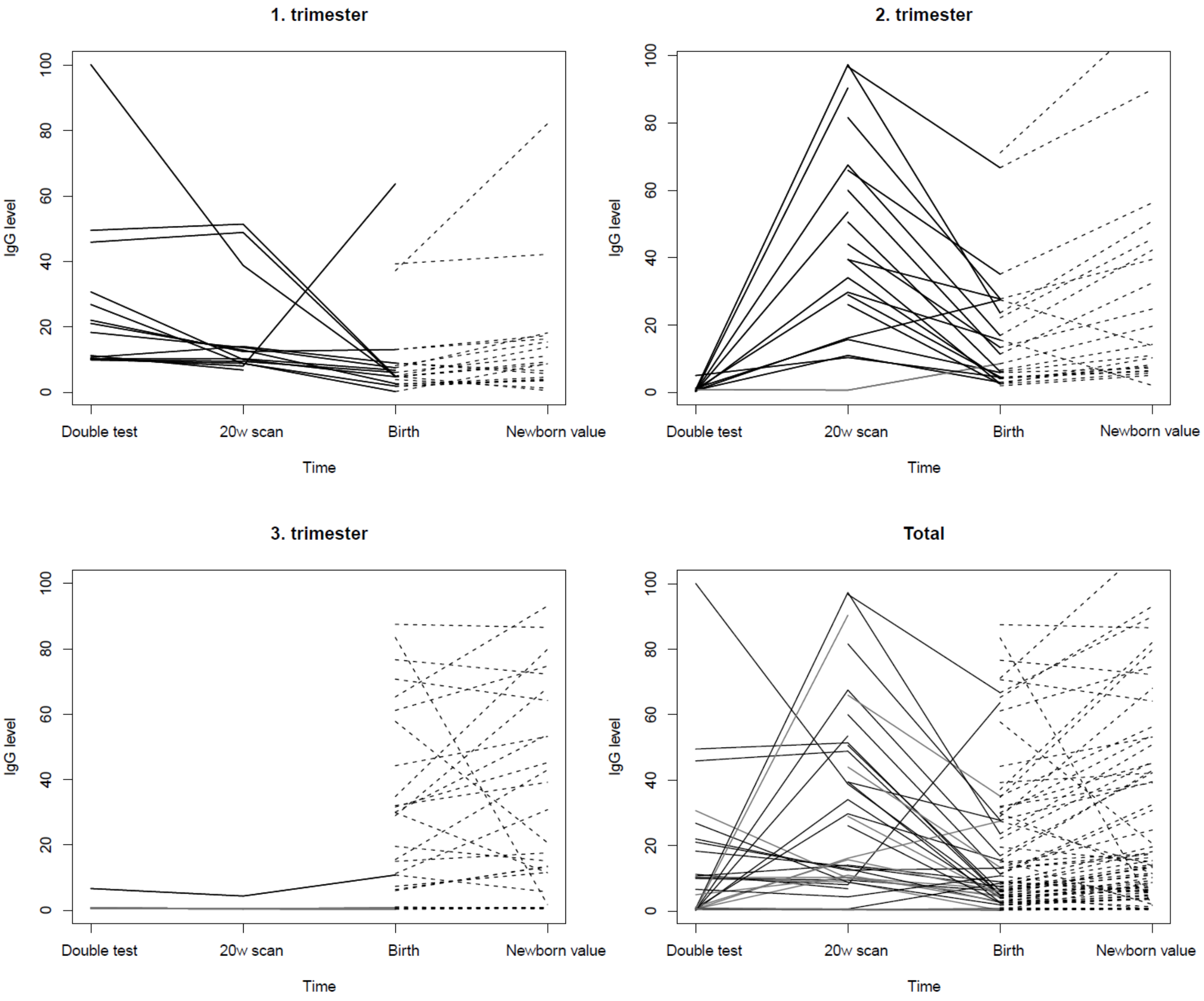

The figure illustrates the level of IgG antibodies for mothers and their newborns in the umbilical cord blood at the time of delivery. The un-dotted line represents the results of the mothers' serology test throughout the pregnancy. The beginning of the dotted line represents the result of the mothers' serology test at birth. The end of the dotted line represents the result of the newborns' umbilical cord serology test. ***1. Trimester)*** *Shows SARS-CoV-2 positive mothers infected in the first trimester with serology test at the first trimester risk assessment test (named: double test), at the second trimester ultrasound and at birth with a correlated serology test from their newborn.* ***2. Trimester)*** *Shows SARS-CoV-2 positive mothers infected in the second trimester with serology test at the second trimester ultrasound and at birth with a correlated serology test from their newborn.* ***3. trimester)*** *Shows SARS-CoV-2 positive mothers infected in the third trimester with serology test at birth with a correlated serology test from their newborn.* ***Total)*** *Includes all SARS-CoV-2 positive mothers with all serology tests taken during pregnancy with their correlating serology test from their newborn.*
